# Carbon fibre/polyether ether ketone (CF/PEEK) implants in orthopaedic oncology

**DOI:** 10.1186/s12957-018-1545-9

**Published:** 2018-12-28

**Authors:** Christoph J. Laux, Sandro M. Hodel, Mazda Farshad, Daniel A. Müller

**Affiliations:** Balgrist University Hospital, Forchstrasse 340, 8008 Zurich, Switzerland

**Keywords:** Orthopaedic oncology, Bone metastases, CF/PEEK, Polyether ether ketone, Radiation therapy, Imaging

## Abstract

**Background:**

Radiation therapy is an important therapeutic element in musculoskeletal tumours, especially when encountering multiple or painful lesions. In osteolytic lesions, a surgical stabilization with implants is often required. However, metallic implants not only complicate the CT-based planning of a subsequent radiation therapy, but also have an uncontrollable dose-modulating effect in adjuvant radiotherapy. In addition, follow-up imaging and the diagnosis of local recurrences are often obscured by metallic artefacts. Radiolucent implants consisting of carbon/polyether ether ketone (CF/PEEK) therefore facilitate adjuvant radiation therapy and follow-up imaging of bone lesions. We hereby present clinical cases with application of CF/PEEK implants in orthopaedic tumour surgery.

**Methods:**

We report a single-centre experience of three selected patients with surgical stabilization of osteolytic bone lesions using CF/PEEK implants. Detailed information about the clinical presentation, preoperative considerations, surgical procedures and postoperative results is provided for each case.

**Results:**

One spinal lesion (T12 vertebral body), one lesion of the upper extremity (humerus) and one of the lower extremities (tibia) were surgically stabilized with use of CF/PEEK implants. With a mean follow-up of 12 months (range 6–25 months), no adverse events were observed. Two patients received adjuvant radiotherapy. Follow-up imaging was obtained in all patients.

**Conclusion:**

The applicability of CF/PEEK implants in orthopaedic tumour surgery is good with respect to postoperative follow-up imaging, application of adjuvant radiotherapy and intraoperative handling. As a result of the unique material properties, oncological patients might particularly benefit from CF/PEEK implants.

## Background

The bone is a frequent site of metastases of a variety of tumours. Radiation therapy is an important element in musculoskeletal oncology, especially when encountering multiple or osteolytic lesions. Often, a surgical stabilization with implants is required. However, metallic implants not only impair the CT-based planning of a subsequent radiation therapy, but also have a dose modulating effect in radiotherapy [1, 2]. When administering radiation therapy, metallic implants affect both the surrounding tissue due to backscattering and inadvertent dose increase and the lesion to be irradiated due to beam attenuation compromising the therapeutic effect. In addition, follow-up imaging and the diagnosis of local recurrences are often obscured by metallic artefacts [3]. Radiolucent implants consisting of carbon fibre reinforced polyether ether ketone (CF/PEEK) therefore facilitate adjuvant radiation therapy and follow-up imaging of bone lesions. To our best knowledge, the only study investigating the beam attenuation conditioned by CF/PEEK implants in a solid water phantom has been published in 2017 by Nevelsky et al. [1]. CF/PEEK pedicle screws have been shown to cause no backscatter effect and only a minimal dose attenuation in contrast to pedicle screws consisting of titanium. The maximum overdose to adjacent tissues due to backscattering accounts for 10% in titanium screws, whereas CF/PEEK screws did not show a backscatter effect at all. Additionally, titanium screws attenuated the radiation beam by 30%, whereas CF/PEEK screws showed only minimal dose alteration with a calculated attenuation of 5%.

With regard to biomechanical aspects, CF/PEEK has been investigated more expansively. By addition of continuous carbon fibres, the elastic modulus of PEEK is raised and can be adapted by the amount and orientation of the carbon fibres. In contrast to stiffer titanium-alloy implants with an elastic modulus of 106 to 155 GPa, available orthopaedic CF/PEEK implants have an elastic modulus close to that of a cortical bone (18 GPa) [4, 5]. More compliant implants reduce stress peaks in the bone implant interface and show improved longevity in vitro [6]. This is particularly desirable in a structurally poor bone, where sufficient bone healing is expected either late or not at all. Thus, more elastic implants are favorable in osteoporotic or pathological fractures and less frequently lead to mechanical complications like screw cut-out or loss of reduction [6, 7]. Overall, the available clinical data is yet somewhat inconsistent regarding the long-term implant reliability [8, 9].

We hereby present clinical cases with application of CF/PEEK implants in orthopaedic tumour surgery [4].

## Material and methods

We report a single-centre experience of three selected patients with surgical stabilization of osteolytic bone lesions in different anatomic regions using CF/PEEK implants. One spinal lesion (T12 vertebral body), one lesion of the upper extremity (humerus) and one of the lower extremity (tibia) were surgically stabilized. Detailed information about the clinical presentation, preoperative considerations, surgical procedures and postoperative results is provided for each case.

## Results

One spinal lesion (T12 vertebral body), one lesion of the upper extremity (humerus) and one of the lower extremity (tibia) were surgically stabilized with use of CF/PEEK implants. With a mean follow-up of 12 months (range 6–25 months), no adverse events were observed. Two patients received adjuvant radiotherapy. Follow-up imaging was obtained in all patients. The bony structures could be assessed without any metal-induced impairments in all imaging studies.

### Case 1

**Fig. 1:**
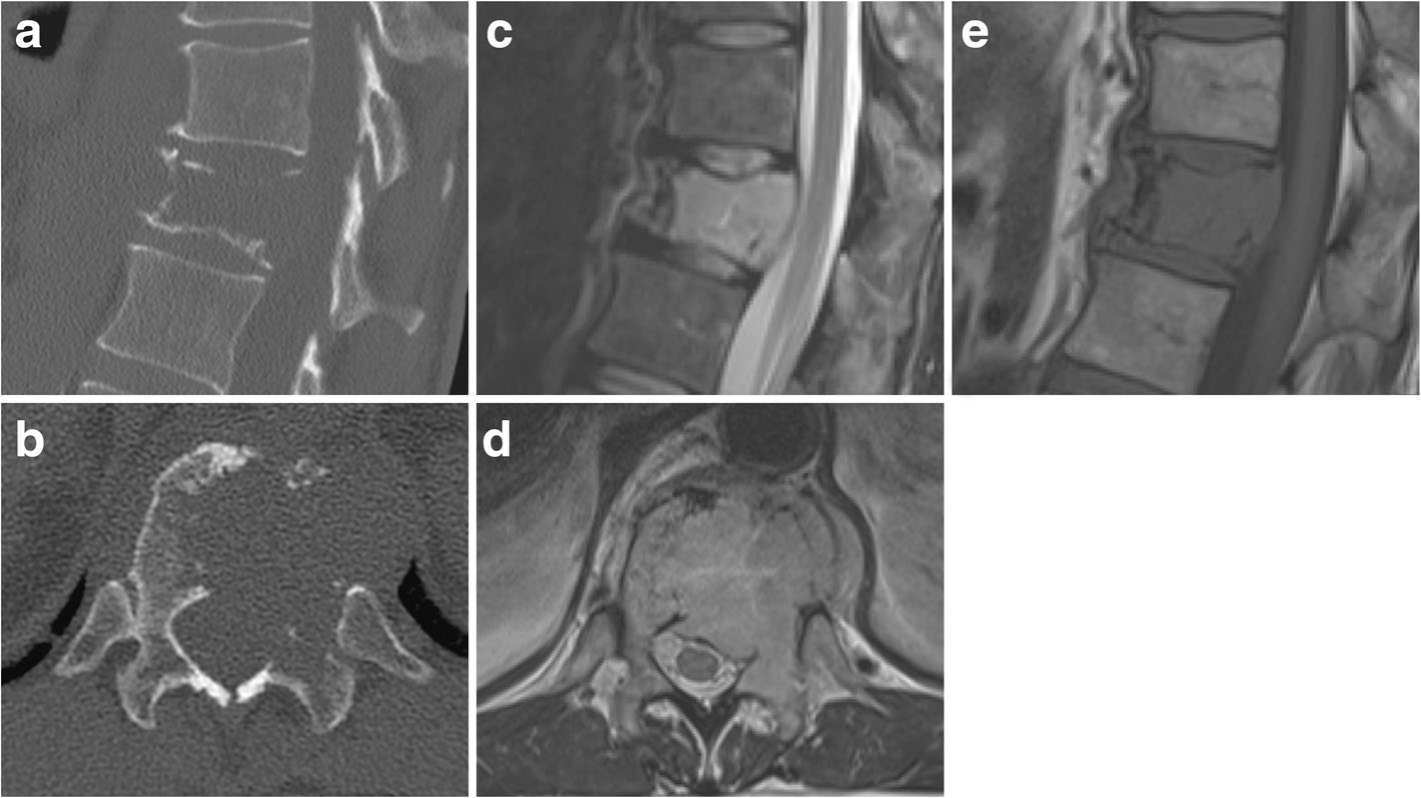
CT scan (**a**, **b**) of the lytic lesion of the T12 vertebra with unilateral posterolateral involvement. MRI with T2-hyperintense and T1-hypointense lesion (**c**, **e**) and more obvious involvement of the posterior elements (**d**)

**Fig. 2:**
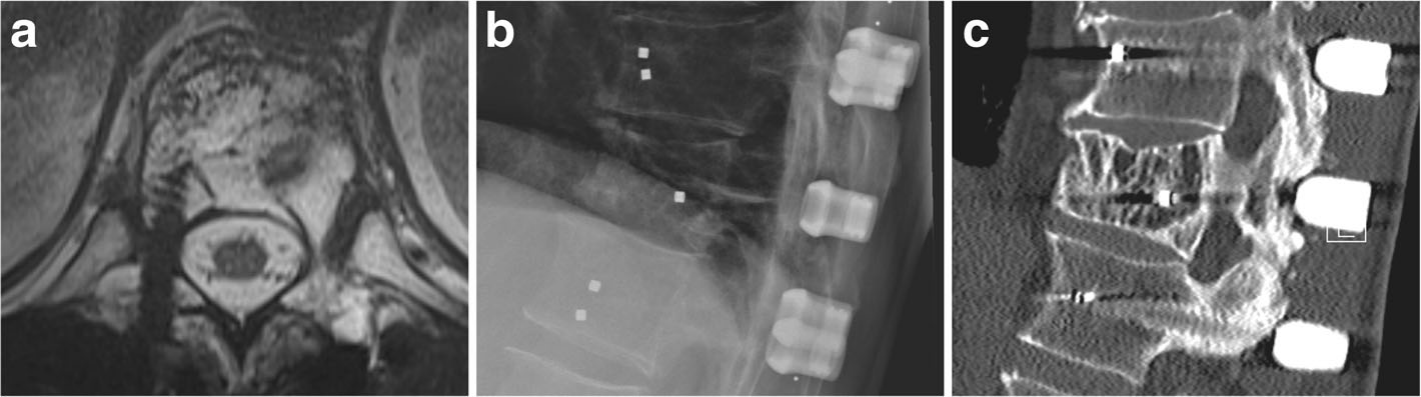
The postoperative imaging provides excellent assessability of the bony anatomy despite proximity of the implants. The implants are best depicted in the MRI with artefact reduction protocol (**a**, **b**). The last follow-up CT scan shows the accomplished posterolateral fusion (**c**)

A 77-year-old male patient presents with severe non-radiating back pain at the thoracolumbar junction without accompanying sensorimotor deficits. Radiological assessment with conventional x-ray and magnetic resonance imaging (MRI) shows an osteolytic lesion with pathologic fracture of the T12 vertebra, unilaterally diminished vertebral body height and consecutive de novo scoliosis of 10° (Fig. 1). The lesion also features a left paravertebral and epidural soft-tissue involvement without compromise of neurological structures. CT-guided transpedicular biopsy revealed multiple myeloma (Durie-Salmon stage I, R-ISS stage I). With a Spine Instability Neoplastic Score (SINS) of 13 points (junctional, non-mechanical pain, lytic lesion, deformity (scoliosis), < 50% collapse, unilateral involvement of the posterolateral elements), the lesion was judged unstable and surgical stabilization was planned prior to subsequent radiation therapy [10]. The patient underwent unnavigated dorsal instrumentation and fusion from T11 to L1 using CF/PEEK pedicle screws and rods (icotec AG BlackArmor® pedicle system 5.5 mm) with apposition of iliac crest autograft and demineralized bone matrix (Fig. 2). Postoperatively, the patient had to wear a supportive customized thoracolumbar orthosis for 8 weeks. After rehabilitation and uneventful wound healing, a consolidating and analgesic radiation therapy with CT-based planning (total 30 Gray) was administered in a volumetric modulated arc therapy (VMAT) technique. A cytotoxic therapy with bortezomib, cyclophosphamide and dexamethasone has been initiated for systemic disease progression and achieved full remission after four cycles. No further surgical intervention was undertaken during the follow-up period (25 months). From a surgical point of view, the patient reported a very satisfactory outcome and stated an Oswestry Disability Index of 20%.

### Case 2

**Fig. 3:**
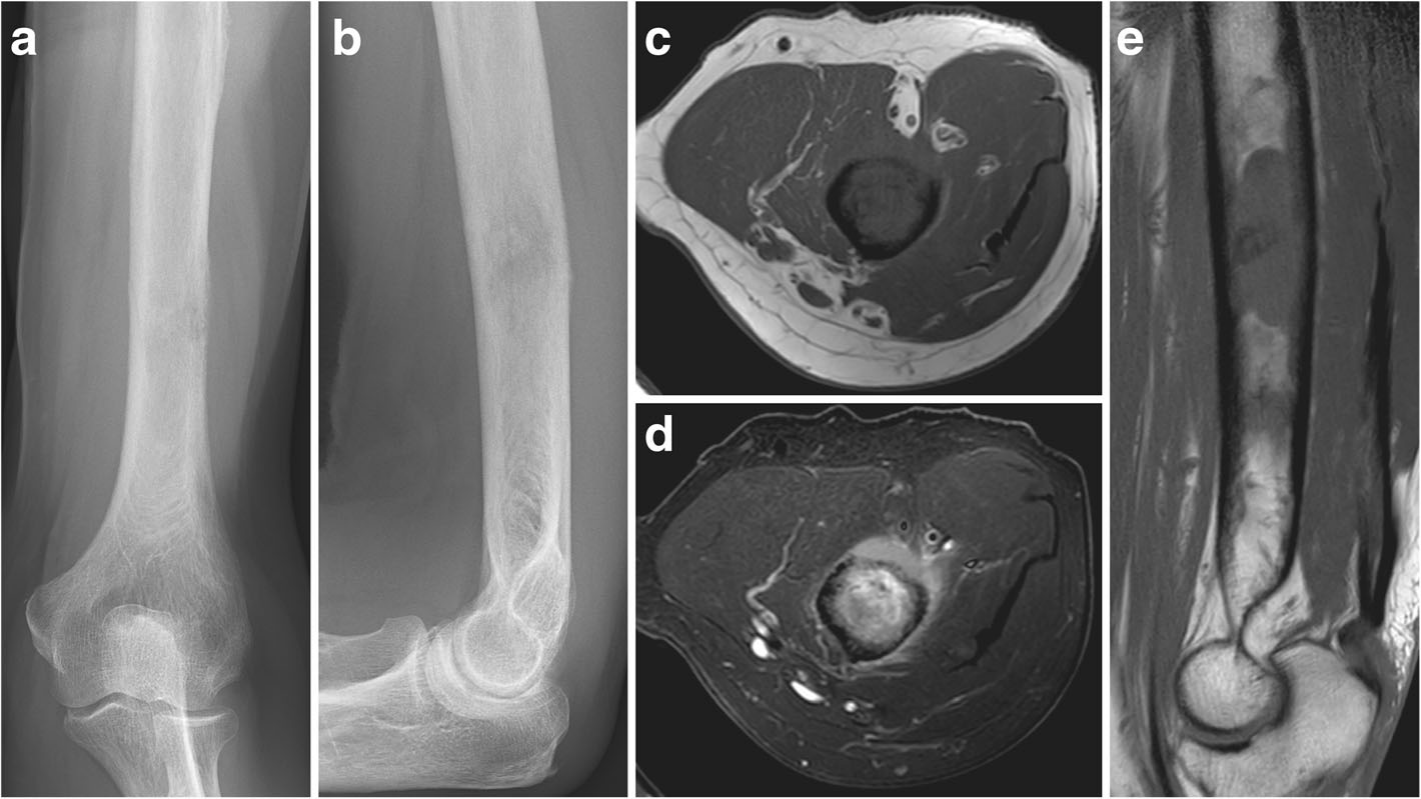
Initial radiographs of the lytic lesion in the distal humeral diaphysis (**a**, **b**). MRI with axial T1 (**c**) and STIR (**d**) sequences of the lytic lesion with significant cortical erosion and perifocal oedema. The sagittal T1 sequence (**e**) shows the longitudinal extent of the lesion

**Fig. 4:**
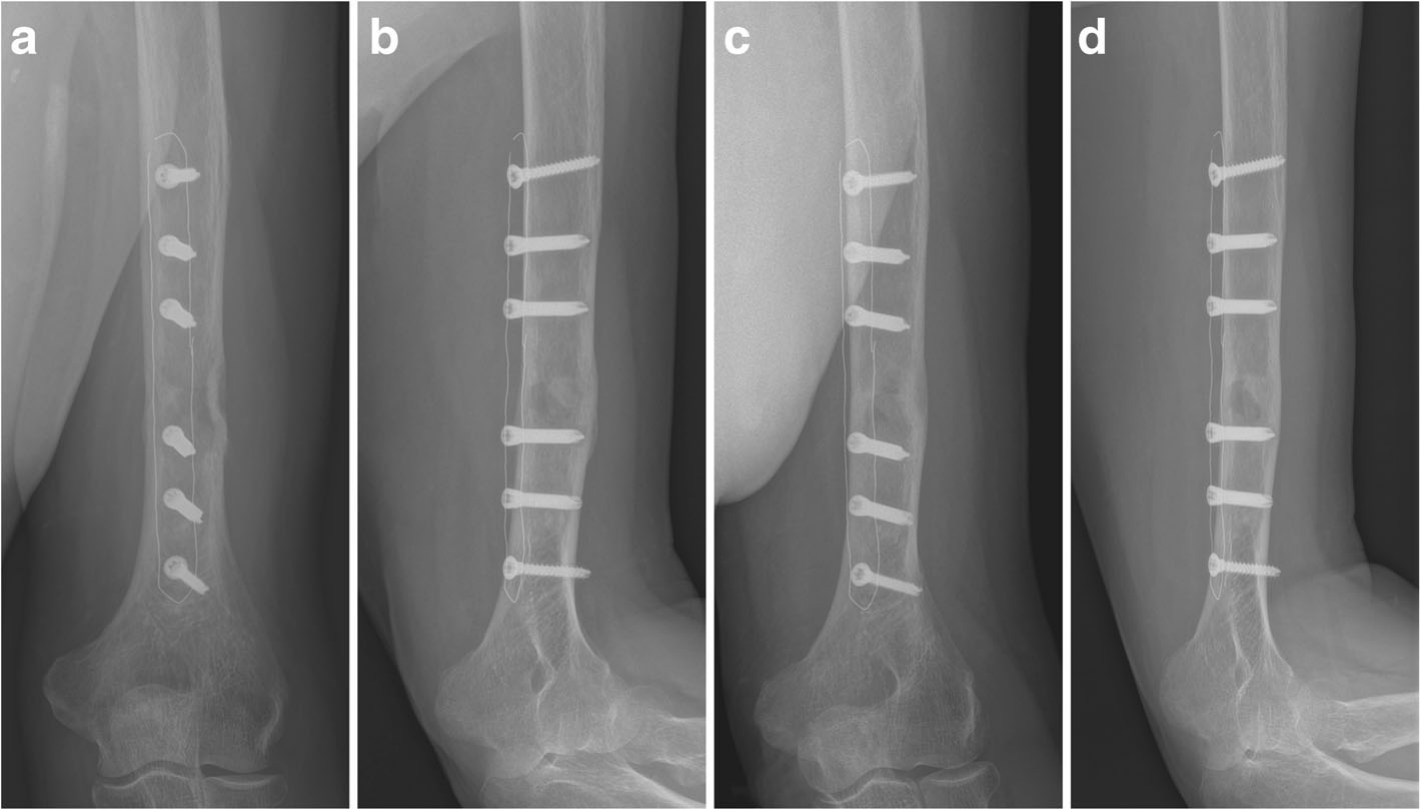
The postoperative x-ray at 6 weeks (**a**, **b**) and 6 months of follow-up (**c**, **d**) shows bridge plating of the lesion and anatomic bone alignment and eventually completed osseous consolidation

A 77-year-old male patient recognizes a mass in his dorsal upper arm. He seeks medical attendance when the lesion starts to be painful during the night a few weeks later. The patient also reports an unintended weight loss of 5 kg and recent night sweats. Radiological imaging shows an osteolytic lesion of the distal humerus with permeative growth and radial cortical penetration (Mirels’ score 8 points [11]) (Fig. 3). Histopathologic evaluation after CT-guided core needle biopsy yielded an extensively necrotic metastasis from a prostatic carcinoma (pT1c cN0 M1b, Gleason Score 4 + 4 = 8, prostate-specific antigen 499 ng/ml). A systemic therapy with denosumab and goserelin was initiated. Due to significant cortical weakening (30%) with a non-displaced pathologic fracture, surgical stabilization was opted for prior to cytotoxic therapy with docetaxel and radiotherapy. Intralesional curettage was followed by open bridge plating using a CF/PEEK 4.5-mm locking compression plate (CarboFix Orthopedics Ltd. “Piccolo” Narrow Diaphyseal Plate) (Fig. 4). The intraoperative handling of the chosen implant was straightforward and without unexpected incidents. Postoperatively, no weight-bearing and only careful passive mobilization was permitted for 6 weeks. Additionally, an arm sling was worn until complete wound healing had been attained. During routine follow-up, the patient presented without pain at his upper arm. The palliative radiotherapy (total 30 Gray) was administered subsequently. Likewise, the remaining follow-up (6 months) was uneventful. With regard to his arm, the patient reported very favourable and pain-free course allowing for any desired sports activity and stating a QuickDASH score of 22.5 points as well as a subjective limb value of 90%.

### Case 3

**Fig. 5:**
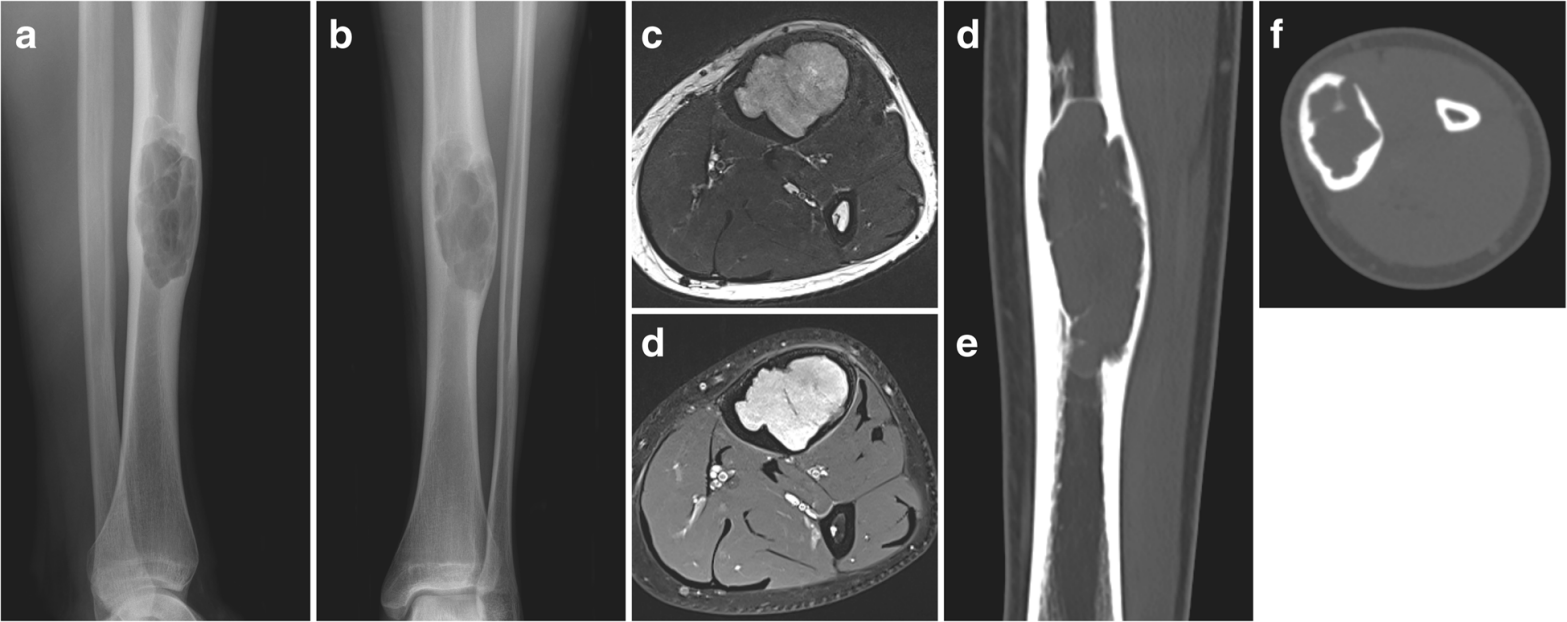
Initial imaging with radiographs (**a**, **b**), axial T2 (**c**) and post-contrast T1 fat-saturated dixon (**d**) sequences as well as CT scans (**e**, **f**) showing the lytic and lobulated aspect of the lesion in the tibial diaphysis with cortical erosion and focal penetration

**Fig. 6:**
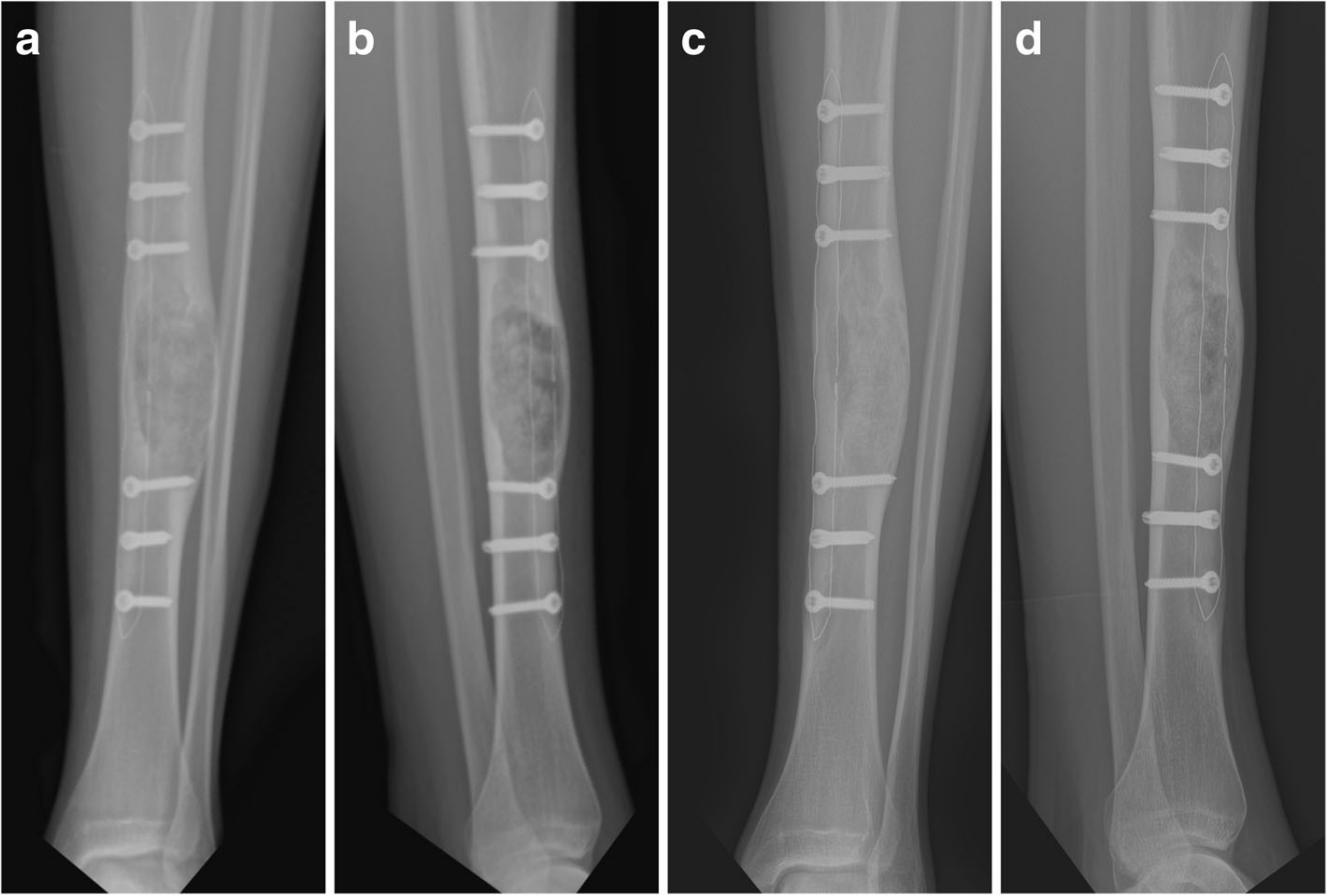
Postoperative radiographs with excellent assessability of bone healing after 6 weeks (**a**, **b**) and 6 months (**c**, **d**)

A 17-year-old male patient experiences intermittent pain and swelling at his left anterior tibia. Four months after onset of symptoms, the patient seeks medical advice. With a radiographically lytic and lobulated lesion of the tibial diaphysis featuring cortical erosion and focal penetration (Fig. 5), referral to our institution was prompted. CT-guided needle biopsy revealed the rare finding of an intraosseous schwannoma. Even though benign, tumour resection was aspired in this symptomatic patient. To allow for better radiological surveillance during follow-up of this rare lesion, a CF/PEEK rather than a metallic implant was chosen. Due to the expansive growth (Mirels’ score 9 points [11]), prophylactic plate stabilization was needed. After complete curettage of the intraosseous lesion, the cavity was filled with cancellous bone allograft. Surgery was then completed by open bridge plating with use of a CF/PEEK 4.5-mm locking compression plate (CarboFix Orthopedics Ltd. “Piccolo” Narrow Diaphyseal Plate) (Fig. 6). Again, the applicability of the chosen CF/PEEK implant was uncomplicated. Besides a prolonged wound secretion without need for special measures, the postoperative course was uneventful. After completing 48 h of relaxed bed rest, the patient was mobilized on crutches with partial weight-bearing (15 kg). During routine follow-up, the patient presented pain free with unremarkable clinical findings. Radiographically, progressive ossification could be detected throughout the follow-up (8 months). At the last follow-up, the patient-reported outcome using the SGOT (Swiss Society of Orthopaedics and Traumatology) Minimal Dataset revealed a pain-free well-being with full work capacity, a moderate limitation in sports activity (5/10 points) and a subjective limb value of 90%.

## Discussion

CF/PEEK is a biocompatible composite material with emerging importance in musculoskeletal surgery. It already has been investigated in spinal surgery [2, 3, 12, 13], but the literature still lacks clinical evidence in musculoskeletal oncology [4]. To our best knowledge, we hereby report the first cases of CF/PEEK plating in orthopaedic tumour surgery [14].

The proposed advantages of CF/PEEK implants are mostly related to their radiolucent properties. CF/PEEK shows significantly less artefacts in computed tomography (CT) as well as magnetic resonance imaging (MRI) and, thus, allows for improved follow-up imaging. Ringel et al. were able to show a significant reduction of the artefact volume on CT and 1.5 Tesla MRI scans in a spinal instrumentation model comparing CF/PEEK pedicle screws to conventional titanium-alloy pedicle screws [3]. Thereby, CF/PEEK implants enhance the radiological follow-up of bone lesions with regard to their healing, progression, or relapse. The reduction of metallic artefacts also improves the CT-based planning of subsequent radiation therapy as radiodensity (given in Hounsfield Units) of CF/PEEK implants is closer to that of the tissue to be irradiated [3].

As a consequence, these characteristics not only affect radiological imaging, but also become very important in radiotherapy. Metallic implants cause significant dose alterations due to beam scattering and attenuation. In their recent experimental study, Nevelsky et al. could quantify the dose perturbation of metallic screws when compared to CF/PEEK screws in a solid water phantom [15]. These characteristics make CF/PEEK implants particularly valuable for patients needing postoperative radiotherapy close to radiosensitive surrounding tissue, especially at the spine. Nevertheless, the magnitude of dose variations within the bone and the implant-bone interface has not yet been quantified and needs further investigation. In addition, it should be borne in mind that—besides the fact that CF/PEEK screws are not readily available on the market—the dose-modulating effect of titanium screws within a CF/PEEK plate and distant to the lesion is yet unknown and probably dependent on the orientation of the radiation beam with reference to the screw orientation. The absence of metallic artefacts in immediate proximity to the lesion presumably is more relevant than metallic implant parts more distant to the future target volume. Furthermore, modern radiation techniques, namely the VMAT technique, allow for significantly higher agreement of calculated and measured dose distributions within the target volume in the presence of metallic artefacts [16].

In contrast to metallic implants, CF/PEEK does not allow plate bending or contouring, which admittedly is less important in preventive stabilization of tumourous lesions where stabilization is achieved in a fixateur interne fashion and interfragmentary compression is usually not aimed for. With respect to intraoperative handling, the application of CF/PEEK implants is basically well comparable to that of conventional titanium implants in orthopaedic tumour surgery.

With regard to the intraoperative handling, radiopaque markers along the plate contour or the screw tip help to achieve a correct implant positioning. However, the diagnosis of implant failure is challenging and often requires sectional imaging, especially in spinal implants.

When choosing CF/PEEK implants, some limitations need to be considered. CF/PEEK implants currently are more expensive when compared to titanium implants. Depending on the desired implant design, CF/PEEK implants are less readily available and sometimes need to be ordered well in advance.

## Conclusion

In our series of three different orthopaedic tumour cases, the clinical applicability of CF/PEEK implants is good with respect to postoperative follow-up imaging, application of adjuvant radiotherapy and intraoperative handling. As a result of the unique material properties, oncological patients might particularly benefit from CF/PEEK implants. However, some limitations as to current implant availability need to be considered. Experimental and comparative clinical studies need to quantify clinical and radiotherapeutic benefits.

## Abbreviations

CF/PEEK: Carbon fibre-reinforced polyether ether ketone
CT: Computed tomography
MRI: Magnetic resonance imaging
PEEK: Polyether ether ketone
SINS: Spine Instability Neoplastic Score
VMAT: Volumetric modulated arc therapy
